# Estimating Sleep-Stage Distribution from Respiratory Sounds via Deep Audio Segmentation †

**DOI:** 10.3390/s25206282

**Published:** 2025-10-10

**Authors:** Seungeon Choi, Joshep Shin, Yunu Kim, Jaemyung Shin, Minsam Ko

**Affiliations:** 1 Department of Applied Artificial Intelligence, Hanyang University, Ansan 15588, Republic of Korea; tmddjs444@hanyang.ac.kr (S.C.); joeshin3956@hanyang.ac.kr (J.S.); ssalmon@hanyang.ac.kr (Y.K.); 2 DelightRoom, Seoul 06611, Republic of Korea; jay@delightroom.com

**Keywords:** audio segmentation, respiratory pattern analysis, sleep stage prediction

## Abstract

Accurate assessment of sleep architecture is critical for diagnosing and managing sleep disorders, which significantly impact global health and well-being. While polysomnography (PSG) remains the clinical gold standard, its inherent intrusiveness, high cost, and logistical complexity limit its utility for routine or home-based monitoring. Recent advances highlight that subtle variations in respiratory dynamics, such as respiratory rate and cycle regularity, exhibit meaningful correlations with distinct sleep stages and could serve as valuable non-invasive biomarkers. In this work, we propose a framework for estimating sleep stage distribution—specifically Wake, Light (N1+N2), Deep (N3), and REM—based on respiratory audio captured over a single sleep episode. The framework comprises three principal components: (1) a segmentation module that identifies distinct respiratory cycles in respiratory sounds using a fine-tuned Transformer-based architecture; (2) a feature extraction module that derives a suite of statistical, spectral, and distributional descriptors from these segmented respiratory patterns; and (3) stage-specific regression models that predict the proportion of time spent in each sleep stage. Experiments on the public PSG-Audio dataset (287 subjects; mean 5.3 h per subject), using subject-wise cross-validation, demonstrate the efficacy of the proposed approach. The segmentation model achieved lower RMSE and MAE in predicting respiratory rate and cycle duration, outperforming classical signal-processing baselines. For sleep stage proportion prediction, the proposed method yielded favorable RMSE and MAE across all stages, with the TabPFN model consistently delivering the best results. By quantifying interpretable respiratory features and intentionally avoiding black-box end-to-end modeling, our system may support transparent, contact-free sleep monitoring using passive audio.

## 1. Introduction

Sleep is an indispensable biological necessity, playing a critical role in maintaining comprehensive physiological and psychological health. Impaired sleep quality or undiagnosed and untreated sleep disorders can lead to profound and detrimental consequences for both physical and mental well-being, contributing to a wide array of chronic diseases [1]. Among the many factors that degrade sleep quality, irregular respiratory patterns—such as sleep apnea, hypopnea, and other forms of disordered respiration—are particularly significant. These patterns are intimately linked with severe sleep-related disorders, including obstructive sleep apnea (OSA) and central sleep apnea (CSA), as well as other chronic conditions like cardiovascular disease and diabetes [2]. Consequently, their early and accurate detection and diagnosis are paramount for effective intervention [3].

According to the comprehensive guidelines of the American Academy of Sleep Medicine (AASM), sleep is meticulously classified into distinct stages: Wake, N1 (light non-REM), N2 (deeper non-REM), N3 (deepest non-REM), and REM (Rapid Eye Movement) [4]. Each of these stages is characterized by specific physiological and neurological signatures, reflecting unique brain activity, muscle tone, and ocular movements. A healthy sleep architecture is defined by a characteristic structure and proportion of time spent in each of these stages; disruptions to this distribution are frequently associated with underlying sleep disorders or other health pathologies [5]. Therefore, a comprehensive assessment of sleep quality necessitates the simultaneous, in-depth analysis of both respiratory patterns and the distribution of sleep stages.

Traditional methods for sleep evaluation, notably polysomnography (PSG), offer unparalleled accuracy in sleep stage scoring and respiratory event detection, serving as the clinical gold standard. However, PSG studies are inherently constrained by their substantial cost, patient inconvenience due to extensive sensor attachment, and limited feasibility for routine, longitudinal, or home-based applications [6]. These practical limitations have spurred a growing academic and industrial interest in developing non-contact, non-invasive sleep monitoring technologies. Earlier approaches primarily relied on classical signal processing techniques [7,8], but these methods often proved susceptible to noise, lacked adaptability to individual physiological variations, and performed poorly in uncontrolled, real-world environments [9].

To overcome these significant challenges, deep learning–based approaches have emerged as exceptionally promising alternatives. These sophisticated models can effectively infer sleep-related conditions and physiological states solely from ambient or tracheal respiratory sounds, enabling critical tasks such as automated snore detection [10,11], robust OSA screening [12,13], and comprehensive sleep stage estimation [14,15]. Furthermore, such approaches elegantly leverage ubiquitous sensing modalities like smartphones and bedside microphones, offering substantial practical benefits including reduced patient burden, enhanced comfort, and the crucial feasibility for scalable daily or nightly monitoring outside of clinical settings [16].

Despite their considerable promise, most existing deep learning studies in this domain tend to adopt an End-to-End framework. These typically involve feeding epoch-level log-Mel spectrogram inputs directly into deep neural networks to classify sleep stages or detect apneic events. While effective for prediction, these models often extract unstructured, implicit features from the entire audio signal. This approach fundamentally lacks the fine-grained, explicit analysis of specific respiratory patterns, such as the precise timing of inhale and exhale phases, or the subtle variations in respiratory cycle length, regularity, and overall variability. Consequently, they are inherently limited in their explainability, making it difficult to discern how specific physiological features contribute to their predictions, thereby hindering clinical interpretation and trust.

The preceding work [17] partially addressed some of these interpretability issues through a deep learning–based audio segmentation model. This model demonstrated superior performance over traditional signal processing methods in accurately segmenting respiratory events even in noisy environments. It enabled the precise extraction of fundamental respiratory indicators, such as respiratory cycles, from sleep respiratory sounds, thereby facilitating a more quantitative analysis of nocturnal respiratory patterns. Unlike other deep models that learn opaque implicit representations, this explicitly tokenizes and analyzes discrete respiratory events, thereby enhancing the interpretability of each segment within a clinically meaningful context. However, its utility has been largely confined to the segmentation of respiratory intervals, falling short of deriving higher-level physiological information directly relevant to sleep architecture.

In this study, we propose a systematic framework that significantly extends the concept of respiratory event segmentation to achieve full sleep-stage distribution estimation. The proposed framework integrates three sequential components:

1 Respiratory Sound Segmentation: High-fidelity segmentation of sleep respiratory sounds into distinct respiratory cycles using a state-of-the-art Transformer-based pre-trained speech model, fine-tuned for this specific task.
2 Respiratory Feature Extraction: Derivation of a diverse suite of signal-based and statistical features from the segmented respiratory events, designed to capture both static and dynamic aspects of respiratory behavior.
3 Sleep-Stage Distribution Prediction: Clinically meaningful estimation of the proportions of time spent in Wake, Light (N1+N2), Deep (N3), and REM sleep stages through a series of dedicated regression models.

By strategically leveraging the well-established correlation between nuanced respiratory dynamics and various sleep stages [18], the proposed framework aims to quantitatively estimate overall sleep stage distributions directly from detailed sleep respiratory patterns. This inherently modular architecture fundamentally enhances both interpretability and explainability compared to conventional End-to-End models. We evaluate the framework on the public PSG-Audio dataset with subject-wise cross-validation.

## 2. Proposed Methods

**Figure 1:**
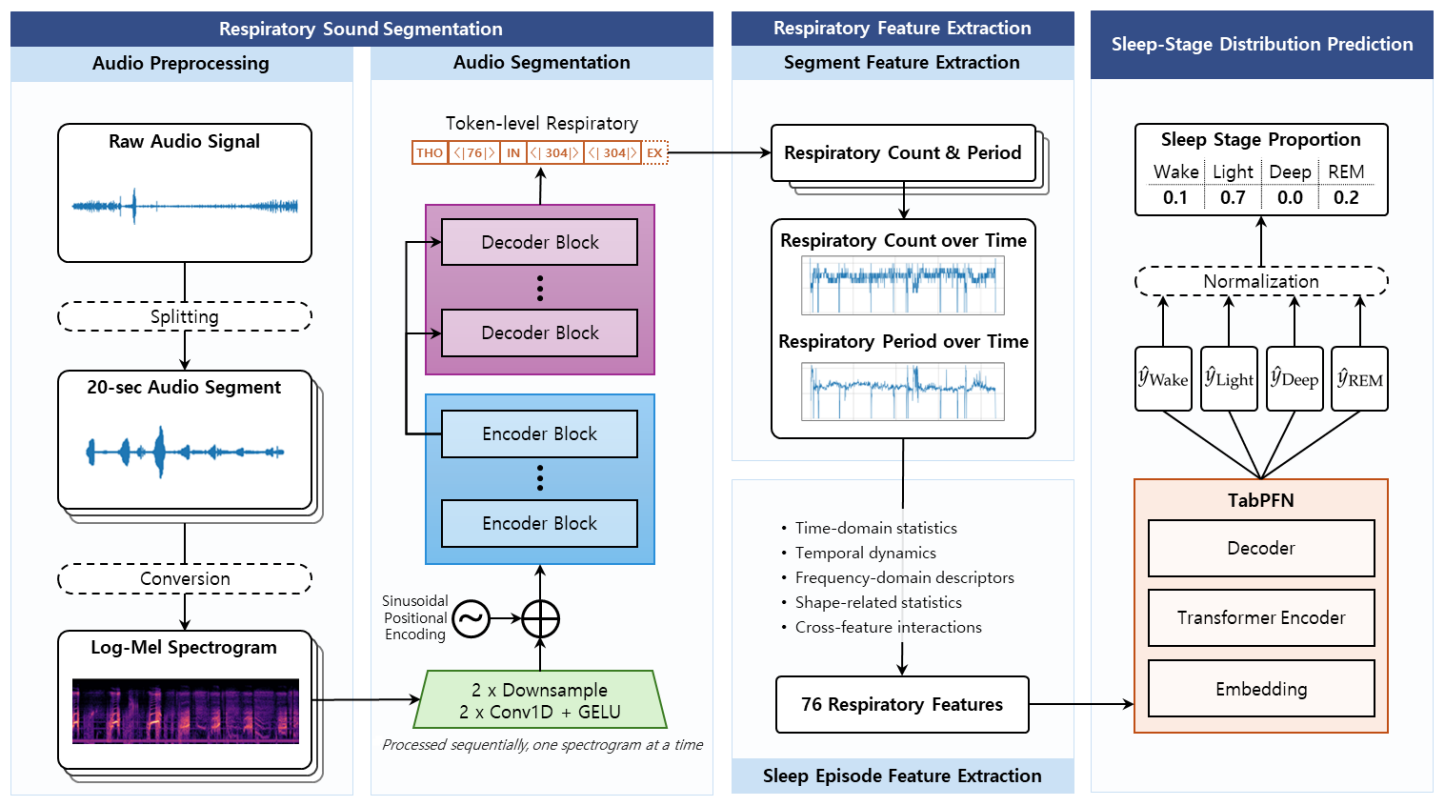
Overall pipeline of the proposed methods.

The proposed approach is specifically designed to analyze sleep respiratory sounds in a modular and inherently interpretable manner. Diverging from a direct End-to-End classification pipeline, we strategically decompose the complex problem of sleep stage proportion estimation into distinct, logically ordered stages that align directly with known physiological processes of respiration and sleep. Figure 1 visually illustrates the overall workflow of the proposed framework and the complete procedure is summarized in Algorithm 1. The framework is meticulously designed with three major components: (1) a Respiratory Sound Segmentation module that accurately detects distinct respiratory cycles from raw respiratory sounds; (2) a Respiratory Feature Extraction module that computes a rich set of statistical and signal-derived metrics from these precisely segmented events; and (3) a Sleep-Stage Distribution Prediction module that estimates the percentage of time spent in each distinct sleep stage.

As summarized in Figure 1, the pipeline decomposes the task into physiologically grounded stages that map raw audio to a subject-level sleep architecture. First, overnight tracheal audio is split into overlapping 20 s windows and converted to log-Mel spectrograms; a fine-tuned Whisper-based Transformer performs frame-wise inhale/exhale/silence classification, with majority voting across overlaps to enforce temporal consistency [19]. Supervision for respiratory onsets is derived from synchronized abdominal effort signals in the PSG-Audio dataset [20]. Second, two window-level respiratory metrics—*Respiratory Count* (inhale onsets per minute) and *Respiratory Period* (median inhale-to-inhale duration)—are computed and then aggregated across the full night into a 76-dimensional, physiologically interpretable feature vector capturing central tendency, dispersion, temporal dynamics, frequency-domain descriptors, and distributional shape. Finally, four stage-specific regressors map this vector to the proportions of Wake, Light (N1+N2), Deep (N3), and REM; the four outputs are normalized to sum to one, yielding a coherent, simplex-constrained estimate of sleep-stage distribution. This modular design makes every step traceable to measurable respiratory quantities and improves clinical interpretability relative to end-to-end spectrogram classifiers.

**Algorithm 1:** Estimating sleep-stage distribution from respiratory audio

**Input**:Overnight tracheal audio a(t); window length W=20 s; hop *H*; pretrained Whisper-based segmentation model $M_{\mathrm{seg}}$; post-processing rules R; feature map Φ(·); per-stage regressors ${\left\{f_{s}\right\}}_{s\in \{\mathrm{Wake},\mathrm{Light},\mathrm{Deep},\mathrm{REM}\}}$
**Output**:Sleep-stage proportion vector $\hat{p}\in R^{4}$, $\sum_{s}{\hat{p}}_{s}=1$



1**Step 1: Spectrogramization and frame-wise segmentation**;2Split a(t) into overlapping windows $\left\{a_{i}\right\}$ of length *W* with hop *H*;3Convert each $a_{i}$ to log-Mel spectrogram $X_{i}$;4Obtain frame posteriors ${\hat{y}}_{i}=M_{\mathrm{seg}}\left(X_{i}\right)$ over {inhale, exhale, silence};5Fuse overlapping predictions by majority voting to get a single label per global frame;

6**Step 2: Event parsing and onset extraction**;7Merge consecutive identical labels into runs; identify candidate transitions {silence,exhale}→inhale;8Apply R (min-duration, hysteresis, refractory) to suppress spurious transitions;9Record inhale-onset timestamps $T={\left\{t_{k}^{\left(\mathrm{inh}\right)}\right\}}_{k=1}^{K}$;

10**Step 3: Window-level respiratory metrics**;11For each window *i*:12$\;\;{\mathrm{RC}}_{i}\leftarrow$ number of onsets in $a_{i}$ (breaths/min);13$\;\;{\mathrm{RP}}_{i}\leftarrow \mathrm{median}\left(t_{k+1}^{\left(\mathrm{inh}\right)}-t_{k}^{\left(\mathrm{inh}\right)}\right)$ using onsets within/near $a_{i}$

14**Step 4: Episode-level feature aggregation**;15Compute feature vector $x\in R^{76}$ from $\left\{{\mathrm{RC}}_{i},{\mathrm{RP}}_{i}\right\}$ via Φ(·)16(e.g., mean, std, IQR, autocorr, spectral entropy, skewness, kurtosis, CV, cross-metrics);

17**Step 5: Stage-wise regression and simplex projection**;18For each stage s∈{Wake,Light,Deep,REM}: ${\tilde{p}}_{s}\leftarrow f_{s}\left(x\right)$;19Normalize to the simplex: ${\hat{p}}_{s}\leftarrow \max\left(\epsilon ,{\tilde{p}}_{s}\right)/\sum_{s^{\prime }}\max\left(\epsilon ,{\tilde{p}}_{s^{\prime }}\right)$ with small ϵ>0;20**return** $\hat{p}=\left[{\hat{p}}_{\mathrm{Wake}},{\hat{p}}_{\mathrm{Light}},{\hat{p}}_{\mathrm{Deep}},{\hat{p}}_{\mathrm{REM}}\right]$;


### 2.1. Respiratory Sound Segmentation

The initial and foundational module precisely segments continuous respiratory audio into distinct respiratory cycles. This is achieved via a fine-tuned version of WhisperSeg [21], a sophisticated Transformer-based model originally developed for robust speech recognition but adapted here for the unique characteristics of respiratory sounds.

#### 2.1.1. Audio Preprocessing

Raw tracheal audio recordings are first preprocessed by splitting them into fixed-duration, overlapping 20 s segments. Each segment undergoes transformation into a log-Mel spectrogram, a dense time-frequency representation of the audio signal. This spectrogram effectively captures the spectral characteristics and temporal evolution of the respiratory sound, serving as the primary input for the downstream segmentation model.

#### 2.1.2. Audio Segmentation Model

Respiratory sound segmentation is formulated as frame-wise multi-class classification. Overlapping 20 s audio windows are converted into log-Mel spectrograms and passed to a fine-tuned Whisper-based Transformer [19,21]. For each frame, the model outputs posterior probabilities over three classes: *inhale*, *exhale*, and *silence*.

Let *N* be the number of frames per segment and C=3 the number of classes. With one-hot labels $y_{i,c}\in \left\{0,1\right\}$ and predicted probabilities ${\hat{y}}_{i,c}$, the cross-entropy loss is minimized:

(1) $$\begin{array}{c} \begin{array}{c} L=-\frac{1}{N}\sum_{i=1}^{N}\sum_{c=1}^{C}y_{i,c}\;\log\;\left({\hat{y}}_{i,c}\right). \end{array} \end{array}$$

Since windows overlap, a given frame may be classified multiple times. We apply majority voting across overlaps to produce a single hard label per frame. This suppresses transient noise and yields temporally smoother label sequences.

Downstream features require discrete respiratory events rather than frame-level labels. We, therefore, convert the smoothed label sequence into a sequence of inhale onsets via a simple post-processing pass: consecutive frames with identical labels are first merged into runs, and a candidate onset is then identified whenever the sequence transitions from {*silence*, *exhale*} to an *inhale* run. We apply minimum-duration constraints and hysteresis to suppress spurious transitions, and impose a short refractory period to prevent duplicate detections. The first frame of each validated *inhale* run is recorded as the onset timestamp $t_{k}^{\left(\mathrm{inh}\right)}$. Although the *exhale* and *silence* predictions are not used directly as features, they stabilize the boundaries and reduce false onsets in noisy segments. This process yields an ordered sequence of inhale-onset timestamps $\left\{t_{k}^{\left(\mathrm{inh}\right)}\right\}$, which serve as the sole input to the subsequent feature extraction module.

### 2.2. Respiratory Feature Extraction

#### 2.2.1. Segment Feature Extraction

Given the inhale-onset sequence $\left\{t_{k}^{\left(\mathrm{inh}\right)}\right\}$ produced by the segmentation module, we derive two core respiratory metrics for each 20 s segment: *Respiratory Count* and *Respiratory Period*. These metrics provide a compact yet physiologically interpretable description of local respiratory dynamics.

##### Respiratory Count

Respiratory Count is defined as the number of inhale onsets detected within a segment:

(2) $$\begin{array}{c} \begin{array}{c} \mathrm{Respiratory}\;\mathrm{Count}=\#\{t_{k}^{\left(\mathrm{inh}\right)}\in \mathrm{segment}\}. \end{array} \end{array}$$

This can be expressed as breaths-per-minute, a standard indicator of respiratory rate. Abnormalities in this rate are associated with sleep-disordered respiratory such as OSA and CSA [22].

##### Respiratory Period

Respiratory Period captures cycle duration by measuring the interval between consecutive inhale onsets:

(3) $$\begin{array}{c} \begin{array}{c} \mathrm{Respiratory}\;\mathrm{Period}=\mathrm{median}\left(t_{k+1}^{\left(\mathrm{inh}\right)}-t_{k}^{\left(\mathrm{inh}\right)}\right). \end{array} \end{array}$$

We use the median to mitigate the effect of transient irregularities or artifacts. Prolonged or highly variable respiratory periods can indicate apneic events or respiratory instability during sleep [23].

Anchoring features on inhale onsets provides a sharp and consistently detectable landmark across subjects and recording conditions, more robust than exhalation offsets which are often diffuse. Although only the inhale timestamps appear in the formulas, the preceding *exhale* and *silence* frame predictions are crucial for reliable onset detection, since they stabilize boundaries and suppress false positives. The resulting metrics form the foundation for higher-level feature aggregation over an entire sleep episode, leading to the comprehensive 76-dimensional respiratory feature vector described in the next subsection.

#### 2.2.2. Sleep Episode Feature Extraction

Following the initial respiratory event segmentation and the computation of per-segment metrics, the subsequent crucial step involves aggregating these granular measurements across the full duration of the sleep episode. This comprehensive aggregation process yields a robust, global respiratory profile that effectively captures both the macro-structural and dynamic aspects of an individual’s nocturnal respiratory behavior.

We compute a comprehensive set of 76 distinct statistical and signal-derived features from the two primary metrics: Respiratory Count and Respiratory Period. These features are meticulously designed to summarize not only central tendencies but also higher-order variations, distributional characteristics, and intricate temporal patterns over the entire recording period. Specifically, the extracted features encompass the following:

- Time-domain statistics: These include the mean (e.g., average respiratory rate throughout the night), standard deviation (quantifying variability in respiratory), maximum and minimum values (identifying extreme events), and the interquartile range (reflecting the spread and central distribution).
- Temporal dynamics: Features like the slope of moving averages (revealing trends in respiratory rate), autocorrelation coefficients (indicating periodicity and predictability of respiratory cycles), and zero-crossing rate (characterizing signal complexity and changes in the respiratory waveform components).
- Frequency-domain descriptors: Metrics such as the dominant frequency (identifying the primary respiratory rhythm) and spectral entropy (quantifying the regularity or randomness of the respiratory signal’s frequency content).
- Shape-related statistics: These include skewness (assessing the asymmetry of the distribution of respiratory durations), kurtosis (indicating the tailedness, which can highlight extreme respiratory events), and the coefficient of variation (providing a standardized measure of relative variability).
- Cross-feature interactions: Derived metrics like ratios and differences between Respiratory Count and Period (e.g., inhale–exhale ratio, which may indicate airway obstruction or altered respiratory effort patterns).

Collectively, these meticulously engineered descriptors form a 76-dimensional feature vector per subject, representing a rich, structured, and physiologically interpretable summary of their entire overnight respiratory patterns. This vector is capable of encoding both rapid, short-term fluctuations (e.g., irregular bursts of respiratory) and crucial long-term stability (e.g., consistency of respiratory cycle lengths).

Critically, each feature within this vector can be directly interpreted physiologically. For example, an increased variability in Respiratory Period might reflect fragmented sleep, transient hypopneas, or even frank apneic events. This highly structured and interpretable representation provides an explicit, explainable basis for the subsequent, downstream prediction of sleep stage proportions.

### 2.3. Sleep-Stage Distribution Prediction

The final stage of the proposed framework involves estimating the proportion of time spent in each distinct sleep stage, utilizing the subject-level respiratory feature vector generated previously. The framework adopts a separate, dedicated regression model trained for each target sleep stage—namely Wake, Light (N1+N2 combined), Deep (N3), and REM.

Let $x\in R^{76}$ denote the comprehensive 76-dimensional input feature vector for a given sleep episode. For each distinct sleep stage s∈Wake,Light,Deep,REM, the objective is to learn an independent regression function $f_{s}:R^{76}\to \left[0,1\right]$ that accurately predicts the proportion of total sleep time spent in that specific stage, i.e., ${\hat{y}}_{s}=f_{s}\left(x\right)$.

The individual outputs from the four independent models ${\hat{y}}_{\mathrm{Wake}},{\hat{y}}_{\mathrm{Light}},{\hat{y}}_{\mathrm{Deep}},{\hat{y}}_{\mathrm{REM}}$ are subsequently normalized to ensure that their sum precisely equals 1.0. This normalization step is critical as it ensures the predictions accurately represent a valid and complete distribution of the entire sleep architecture.

**Figure 2:**
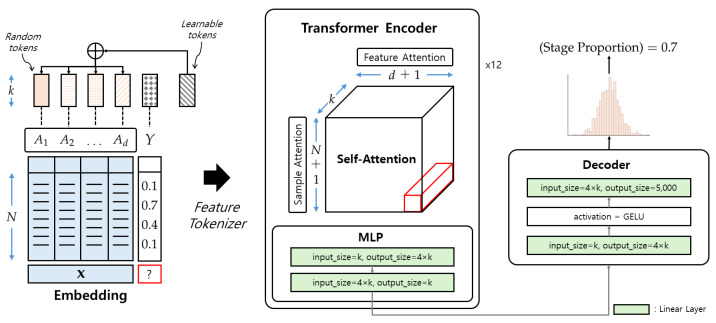
Brief schematic of TabPFN [24]. Illustration adapted from [25].

We adopt TabPFN [24] as our primary regressor (see Figure 2). TabPFN is a Transformer-based foundation model for tabular prediction that performs *amortized inference* using a learned prior over synthetic training tasks, yielding strong accuracy and data efficiency on small-to-medium tabular datasets without extensive hyperparameter tuning. Concretely, TabPFN jointly embeds support samples $\left(x_{i},y_{i}\right)$ and query features $x_{j}$, and performs in-context learning within a 12-layer Transformer encoder, where each layer sequentially applies feature-wise attention, sample-wise attention, and an MLP sublayer, with constraints preventing query samples from attending to each other. After these layers, an additional linear transformation maps the outputs to a piece-wise constant predictive distribution, from which point estimates such as ${\hat{y}}_{j}$ can be derived. This architecture captures nonlinear feature interactions and target dependencies while remaining comparatively robust under limited sample sizes—an appealing property in physiological studies with subject-wise evaluation. For comparison, we also evaluated Random Forest, Support Vector Regression, Linear Regression, Decision Tree Regressor, k-Nearest Neighbors, and a shallow Multi-Layer Perceptron under the identical cross-validation protocol.

Each of these models is independently trained on the same rich respiratory feature input vector but is specifically optimized for a different scalar target—the proportion of time in a particular sleep stage. This strategic approach allows for stage-specific specialization, which is particularly advantageous given that the underlying respiratory correlates may differ significantly across stages (e.g., the characteristic irregularity during REM sleep versus the pronounced stability during Deep sleep).

We emphasize that this is a per-stage regression approach: for each stage *s*, a separate and distinct model is trained using the common 76-dimensional input vector x and a scalar target $y_{s}$. The full set of predictions is then normalized to produce a complete and coherent proportion vector representing the entire sleep architecture. This design significantly improves modularity and allows for selective model optimization based on the unique characteristics of each sleep stage.

## 3. Experimental Setup

### 3.1. Overview

To rigorously validate the performance of the proposed system, we conducted two distinct sets of experiments, each meticulously designed to correspond to its core functional components. The first experiment meticulously evaluates the accuracy of respiratory event segmentation and the precision of the derived respiratory metrics from sleep audio. The second critically assesses the overall effectiveness of the system in predicting comprehensive sleep-stage distribution based on the aggregated, interpretable respiratory features. All experiments were implemented in Python 3.11 with PyTorch 2.5.1 (CUDA 12.1, cuDNN 9.1.0) on Ubuntu 20.04, and training was performed on a single NVIDIA’s A6000 GPU (48 GB VRAM) with an Intel’s Xeon W-2295 @ 3.00 GHz CPU and 64 GB RAM (both from Santa Clara, CA, USA).

### 3.2. Dataset

For all experimental tasks, we utilized the comprehensive PSG-Audio dataset [20]. This publicly available dataset is particularly well-suited for our study as it contains meticulously synchronized multimodal recordings from a diverse cohort of 287 subjects. Each subject’s recording spans an average duration of 5.3 h, providing extensive, real-world data for robust analysis. The dataset specifically includes the following:

- Tracheal sleep audio recordings: Passive acoustic data captured from a microphone placed near the trachea, providing direct information about respiratory sounds.
- Abdominal respiratory effort signals: Physiologically validated signals obtained from a sensor placed on the abdomen, serving as a gold-standard reference for individual respiratory cycles and events.
- Expert-labeled sleep stages (hypnograms): Derived from full polysomnography (PSG) by certified sleep technologists according to AASM guidelines, providing the ground truth for sleep architecture analysis.

This synchronized multimodal information is predominantly leveraged for the development of sleep apnea and snoring detection algorithms [26,27]. The rich and synchronized nature of this dataset is crucial for both training the respiratory segmentation model with precise ground truth and for evaluating the sleep stage proportion predictions against clinical standards.

### 3.3. Task 1: Respiratory Sound Segmentation

The first experimental task is dedicated to thoroughly evaluating the accuracy of segmenting continuous sleep respiratory audio into its fundamental respiratory events, specifically, inhale, exhale, and silence intervals. Furthermore, it assesses the precision of the crucial respiratory metrics directly derived from these segmentations: the respiratory rate (Respiratory Count) and the respiratory cycle duration (Respiratory Period). Both are physiologically meaningful and clinically vital indicators of respiratory function during sleep.

For this task, we systematically segmented the tracheal audio recordings from the PSG-Audio dataset into overlapping 20 s windows using a sliding window approach, resulting in approximately 228,000 such audio segments used for model training and evaluation.

To construct the robust ground truth annotations for respiratory event segmentation, we leveraged the synchronized abdominal respiratory effort signals from the PSG-Audio dataset. We first applied a moving average filter (with a window size of 25 samples) to smooth the abdominal effort signal, thereby effectively reducing high-frequency noise and motion artifacts. Subsequently, we applied the well-established findpeaks algorithm [28] to precisely identify local maxima and minima within the smoothed signal. These points accurately correspond to the physiological onset of inhalation and exhalation, respectively. These precise temporal markers were then meticulously mapped to frame-level labels in the audio timeline, serving as the definitive supervision targets for our segmentation model.

The core segmentation model is founded upon Whisper-Large [19], a powerful Transformer-based architecture initially developed for robust speech recognition. We performed extensive fine-tuning of this pre-trained Whisper model. The log-Mel spectrograms of the segmented audio served as input, with the primary objective being the accurate classification of each audio frame into one of three distinct classes: inhale, exhale, or silence. To mitigate the inherent noisy predictions that often occur at frame boundaries and to ensure temporal consistency, we judiciously applied a majority voting strategy across overlapping frames in the output.

To establish a robust benchmark for the performance of our proposed model, we implemented two widely recognized traditional signal processing baselines. The first method utilizes the Fast Fourier Transform (FFT) to estimate dominant respiratory frequencies. In this approach, raw audio signals are first meticulously filtered, dynamically compressed, and then smoothed using a Gaussian kernel before undergoing spectral analysis. The prominent frequency components are then directly employed to infer respiratory cycles [29]. The second method is based on smoothed peak detection within the energy envelope of the respiratory sound. Inspired by [30], this technique initially transforms the audio into a log-Mel spectrogram, subsequently computes an intensity envelope, and then applies Gaussian smoothing followed by a refined peak detection algorithm. To further improve alignment with precise physiological events, the initially detected peaks are refined using a BFGS optimization approach.

The entire dataset was randomly and rigorously split into training and evaluation sets at a 9:1 ratio, critically ensuring a subject-independent basis. This prevents data leakage and ensures the model’s ability to robust to new, unseen individuals. The model was trained for a substantial 154,900 steps within a single epoch, optimizing for cross-entropy loss. This meticulous fine-tuning procedure successfully adapted the generic yet powerful Whisper model to the highly specialized task of respiratory event segmentation in noisy, real-world sleep audio environments.

For comprehensive performance evaluation, we focused on two crucial downstream metrics derived directly from the segmentation output: Respiratory Count and Respiratory Period. Respiratory Count is precisely defined as the number of inhalation onsets per minute, directly equivalent to the respiratory rate. Respiratory Period is computed as the median interval between consecutive inhale onsets, thereby accurately capturing the duration of the respiratory cycle. These predicted values were then rigorously compared against those meticulously computed from the ground-truth sensor annotations. We quantified performance using Root Mean Squared Error (RMSE) and Mean Absolute Error (MAE). RMSE, being particularly sensitive to larger errors, is especially suitable for evaluating clinical applicability, especially in segments characterized by irregular or disordered respiratory, while MAE provides a direct measure of average prediction deviation.

### 3.4. Task 2: Sleep-Stage Distribution Prediction

The second experimental task aims to precisely estimate the overall distribution of macro sleep stages across a subject’s full night of sleep, relying exclusively on features derived from their continuous nocturnal respiratory sounds. Consistent with prior work on wearable- and audio-based staging, we adopt a four-class mapping: Wake, Light (N1+N2), Deep (N3), and REM [14,31,32]. The original PSG-Audio dataset provides expert-labeled hypnograms according to AASM guidelines, in which each 30 s epoch is annotated as Wake, N1, N2, N3, or REM. For the purpose of this study, we consolidated the two light non-REM stages (N1 and N2) into a single Light category, yielding four macro stages in total. This aggregation is widely adopted in prior research because N1 and N2 share similar physiological characteristics, and distinguishing them reliably from non-EEG modalities such as audio is challenging.

In this specific setting, our objective is to predict the proportion of total sleep time spent in each of the four stages, rather than performing short-term epoch-by-epoch classification. To generate the robust predictive features, we systematically utilized the output from the precisely executed respiratory event segmentation module (Task 1). Specifically, we calculated the Respiratory Count and Respiratory Period for each 20 s audio segment across the entire night. These granular metrics were then meticulously aggregated across the complete sleep session to form a comprehensive, subject-level representation of an individual’s nocturnal respiratory behavior. From each subject’s full sleep episode recording, we extracted a total of 76 distinct features as described in Section 2.2.

For this task, the ground truth sleep stage proportions were derived directly from the expert-labeled sleep stages provided within the PSG-Audio dataset. The total duration of each subject’s recording was first determined, and then the accumulated time spent in Wake, Light, Deep, and REM stages was calculated. These accumulated times were subsequently converted into proportions of the total recording time, serving as the scalar target values for our regression models.

To identify the most effective modeling approach, we compared seven regressors: Linear Regression (LR), Decision Tree Regressor (DTR), Random Forest Regressor (RFR), Support Vector Regression (SVR), k-Nearest Neighbors (kNN), Multi-Layer Perceptron (MLP), and TabPFN [24]. The first six models were implemented with scikit-learn [33] using the hyperparameters listed in Table 1, while TabPFN—a pretrained Transformer-based foundation model for tabular data—was used with its default pretrained weights. This suite spans linear, tree-based, kernel, instance-based, neural, and foundation-model approaches, providing a balanced basis to evaluate accuracy and data efficiency on our 76-feature representation.

To ensure a robust and unbiased evaluation of generalizability, we employed 5-fold subject-wise cross-validation. In each fold, the regressors were trained on data from 80% of the subjects and rigorously tested on the remaining 20%, meticulously ensuring that no subject appeared in both the training and test sets. Model performance was then meticulously averaged across all five folds to provide a reliable assessment of generalizability to unseen individuals.

For evaluation, we computed the Root Mean Squared Error (RMSE) and Mean Absolute Error (MAE) between the predicted and true sleep stage proportions for each of the four stages. These metrics provide direct, quantitative insight into the model’s capability to accurately approximate clinically meaningful sleep architecture solely based on comprehensive respiratory patterns.

## 4. Results

### 4.1. Task 1: Respiratory Sound Segmentation

#### 4.1.1. Segment-Level Quantitative Evaluation

Our initial assessment focused on the accuracy of the model in predicting key respiratory metrics on a per-segment basis, using 20-second audio segments as the input. Each segment was annotated with two critical respiratory features derived from the reference abdominal effort signal: Respiratory Count, representing the number of inhalations per minute and Respiratory Period, the median inhale-to-inhale cycle duration in seconds. These metrics were computed from both the model’s predictions and the ground-truth annotations, and their agreement was quantified using Root Mean Squared Error (RMSE) and Mean Absolute Error (MAE).

Table 2 summarizes the compelling results, directly comparing our Transformer-based segmentation model against two established classical baselines: an FFT-based frequency analysis method and a Peak-Finding method based on smoothed intensity envelopes. Across both evaluation metrics, our proposed model consistently achieved significantly lower errors. Specifically, it recorded an RMSE of 1.44 and an MAE of 0.96 in respiratory count estimation, representing a substantial improvement. For respiratory period estimation, it achieved an RMSE of 1.25 and an MAE of 0.81, further demonstrating its superior performance.

These quantitative results unequivocally confirm that the proposed model not only robust exceptionally well to unseen data but also provides superior, finer-grained segmentation accuracy. This is particularly evident under challenging conditions such as noisy or irregular respiratory, where traditional heuristic-based methods frequently exhibit significant performance degradation.

#### 4.1.2. Sleep Episode Level Prediction

To ascertain the long-term reliability and consistency of the proposed model over extended overnight recordings, we next evaluated the agreement between predicted and ground-truth respiratory statistics aggregated at the individual subject level. For each subject in the held-out test fold (10% of 287), both the average and standard deviation of Respiratory Count and Respiratory Period were computed across their entire sleep session and then rigorously compared against the corresponding sensor-derived ground-truth values.

**Figure 3:**
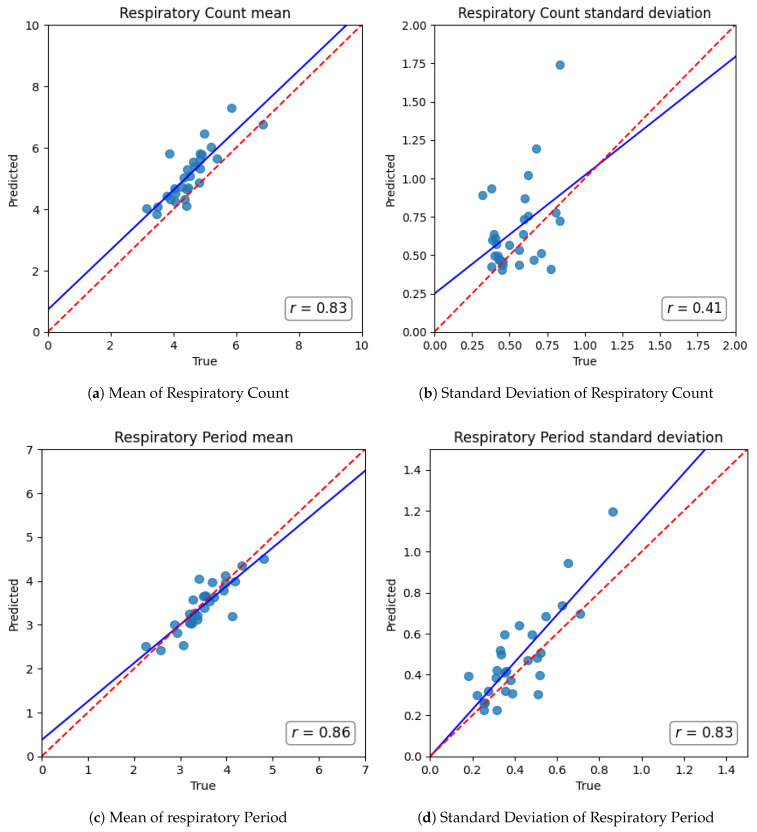
Scatter plots of respiratory metrics at the sleep episode level.

Figure 3 presents compelling scatter plots illustrating the strong correlation between predicted and ground-truth values across test subjects. Robust linear correlations were observed for both metrics: Pearson’s correlation coefficient was r=0.83 for the average Respiratory Count and r=0.86 for the average Respiratory Period. Furthermore, to assess the model’s ability to capture intra-subject variability, we also examined the prediction performance for standard deviation values. The resulting correlations were r=0.41 for the Respiratory Count standard deviation and r=0.83 for the Respiratory Period standard deviation.

These results demonstrate that the model not only captures momentary respiratory events but also reliably tracks both the central tendency and dispersion of respiratory dynamics, enabling robust estimation of representative nocturnal respiratory behavior at the subject level.

**Figure 4:**
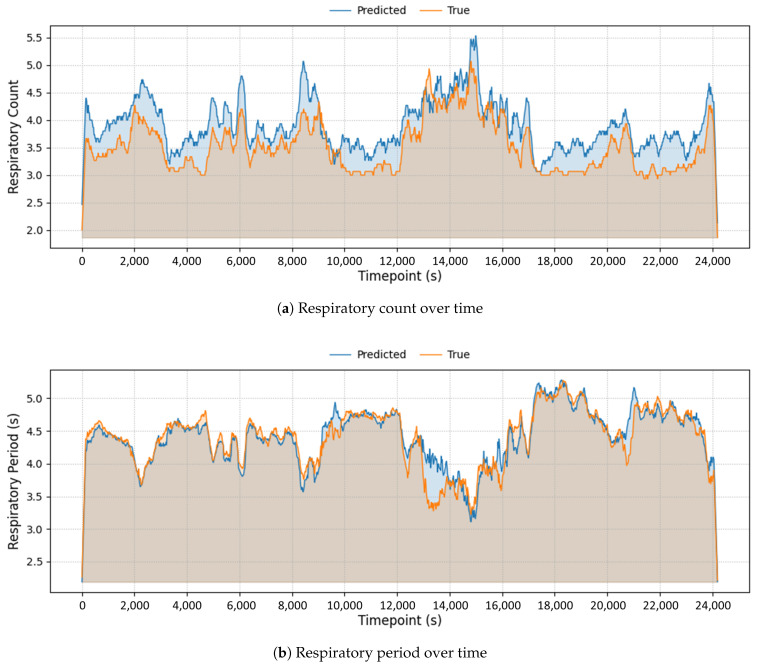
Example of temporal prediction of respiratory metrics over a full sleep episode.

To qualitatively assess the temporal tracking performance of the proposed model, we visualized the predicted respiratory metrics over time for a representative subject across their entire sleep episode. As illustrated in Figure 4, the model’s predictions closely follow the ground-truth trajectories for both Respiratory Count and Respiratory Period, each computed over successive 20 s segments throughout the night.

The time-series plots reveal that the model successfully captures both gradual trends and short-term fluctuations in respiratory dynamics, including sustained increases, periodic plateaus, and abrupt transitions potentially corresponding to respiratory irregularities or stage changes. Importantly, the model demonstrates consistent alignment with the reference signals even during noisy or irregular segments, underscoring its robustness to real-world signal variability.

This subject-level example highlights the model’s capability to provide temporally fine-grained, physiologically meaningful respiratory metrics with high fidelity over extended overnight recordings—an essential quality for real-world applications such as at-home sleep tracking or longitudinal clinical respiratory monitoring.

### 4.2. Task 2: Sleep Stage Distribution Prediction

Table 3 presents the RMSE and MAE scores for each model across the four target sleep-stage proportions (Wake, Light = N1+N2, Deep, REM) under subject-wise 5-fold cross-validation. Across all stages, TabPFN consistently demonstrated superior performance, achieving the lowest RMSE/MAE compared to RF, SVR, and MLP. For instance, for *Wake* TabPFN recorded RMSE/MAE of 0.0583/0.0430; for *Light* (typically the largest proportion of total sleep time) 0.0893/0.0690; for *Deep* 0.0761/0.0435; and for *REM* 0.0560/0.0391. These results suggest that the respiratory features captured by our system are particularly informative for *Deep*—a physiologically distinct and stable stage—while maintaining high accuracy even for *REM*, which is known to exhibit unique and variable respiratory patterns. The other regressors also perform competently but are consistently outperformed by TabPFN, highlighting the effectiveness of its Transformer-based inductive bias for tabular physiological data even with a relatively small number of subjects.

To translate error magnitudes into clinically interpretable criteria, Table 4 reports the fraction of subjects whose absolute percentage error falls within fixed tolerances of ±5/±10/±20 percentage points (pp). At these practical thresholds, the model covers a large majority of cases: on average, 87.85% of subjects fall within ±10 pp, with particularly strong performance for *Deep* (89.79%) and *REM* (91.55%). Stage-wise differences are expected—*Light* shows lower fixed-tolerance accuracy than *Deep*/*REM*, reflecting broader physiological variability and label heterogeneity in N1+N2.

**Figure 5:**
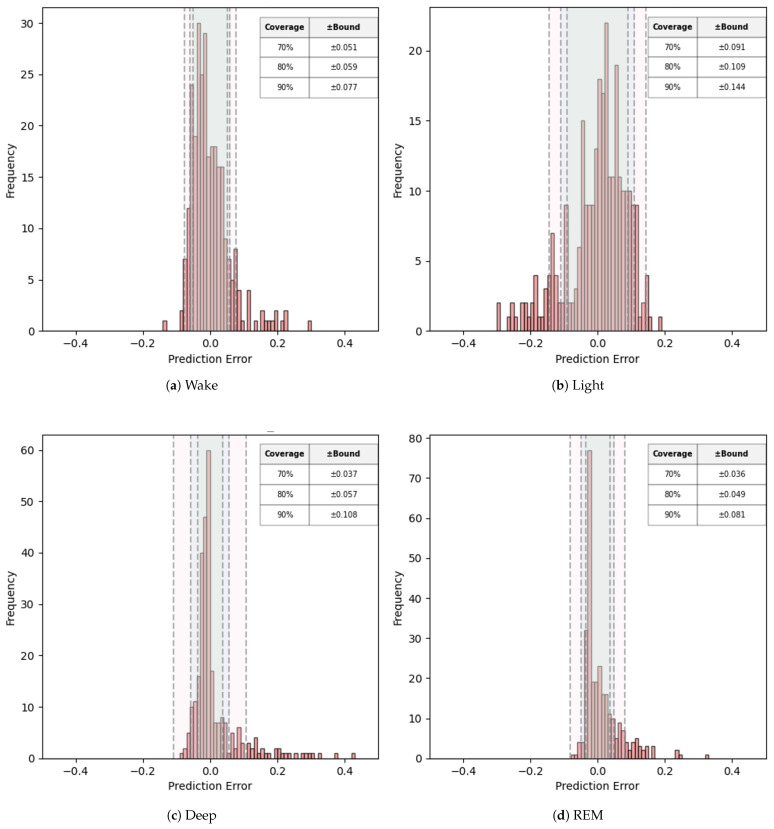
Error distributions of sleep stage distribution predictions.

Complementing this fixed-tolerance view, Figure 5 analyzes the empirical error distributions using a *coverage–bound* perspective that asks the inverse question: *what tolerance is required to achieve a given coverage?* Let $e=\hat{p}-p$ (in pp). For a target coverage c∈{70,80,90}, we compute the smallest symmetric half-width $b_{c}$ such that at least c% of absolute errors lie within $\pm b_{c}$. Figure 5 shows per-stage histograms with vertical dashed lines at $\pm b_{c}$ (the shaded band highlights the c=80% case). For example, an 80% coverage ±Bound of ±0.057 for Deep indicates that 80% of subjects have Deep-percentage errors within ±5.7 pp. Taken together, Table 4 answers *what coverage is achieved at a given tolerance*, while Figure 5 answers *what tolerance is required for a target coverage*; both views are consistent with the tighter, near-zero-centered error distributions observed for Deep/REM and their lower RMSE/MAE under TabPFN.

Finally, Figure 5 also visualizes the close alignment between the *predicted* and *ground-truth* distributions of stage proportions, confirming that the models recover not only subject-level proportions but also the overall balance of stages across the cohort. Slight deviations for *Wake* and *Deep* suggest avenues for future refinement (e.g., additional physiological channels or stage-specific feature engineering).

## 5. Discussion

### 5.1. Clinical and Physiological Implications

This study demonstrates that subtle respiratory sound patterns carry significant information about sleep architecture, enabling accurate and non-intrusive sleep monitoring. By explicitly segmenting and analyzing respiratory cycles, our framework achieves a level of performance and interpretability that favorably compares with prior audio-based sleep staging approaches. Crucially, our findings reinforce long-standing observations that respiration changes with sleep depth – for instance, respiratory tends to become slower and more regular in deep N3 sleep, whereas it is more irregular during REM and wakefulness [34]. Our system effectively exploits these physiological differences to yield robust estimates of overall sleep stage composition directly from respiratory sounds.

The high fidelity of our respiratory segmentation module is fundamental to the framework’s success. Our fine-tuned Transformer model accurately distinguished inhale/exhale phases, even in realistic, noisy conditions, significantly outperforming classical signal-processing methods. The marked reduction in error for both respiratory rate and cycle duration indicates that deep learning can overcome limitations of earlier heuristic approaches, which often required high-quality audio or close-contact sensors to function reliably [14]. This advance is crucial as it ensures that downstream features accurately reflect physiological respiratory rather than artifacts. Practically, the ability to track respiratory rate and variability with sub-minute resolution means our system can capture transient respiratory irregularities that might indicate sleep instability or arousals. This granularity was difficult to attain with traditional methods that often faltered in low signal-to-noise settings [14]. Our approach, by contrast, maintained accuracy across a full night, suggesting robust handling of real-world respiratory variability.

Building on this solid respiratory foundation, the sleep stage proportion estimator yielded promising accuracy. The model’s predictions for time spent in Wake, Light, Deep, and REM sleep were within only a few percentage points of the true values, which is notable given the use of a single audio modality. The particularly low error for Deep sleep is significant and likely stems from the distinctive respiratory signatures of slow-wave sleep—characterized by pronounced regularity and slower respiratory [34]. In essence, our findings confirm that respiratory sound alone is a strong proxy for sleep architecture, echoing earlier studies that different sleep stages manifest unique sound and respiratory profiles [35]. The ability to estimate sleep architecture from audio could have substantial clinical value; for example, capturing reductions in REM or Deep sleep proportion (often associated with aging or disorders) no longer necessitates full PSG but could potentially be achieved with a bedroom microphone.

Moreover, by outputting an easily interpretable summary of sleep (percentages of each stage), our system aligns with how clinicians and sleep researchers typically evaluate sleep quality—through stage distribution and sleep efficiency rather than raw epoch-by-epoch labels. While minute-by-minute sleep stage classification models can also derive these proportions by aggregating epoch-level predictions over a full night, most prior end-to-end approaches offer limited insight into why a certain stage was predicted for a given epoch, as they often rely on black-box mappings from sound to labels [14]. In contrast, our framework’s predictions can be directly traced back to concrete, physiologically meaningful features, such as a higher average respiratory rate or greater variability in respiratory period. This explicit link significantly enhances trust and transparency, which is particularly vital in medical contexts, allowing clinicians to understand the physiological basis of the assessment.

### 5.2. Technical Advances of Transformer-Based Segmentation

To better contextualize these improvements, we highlight the specific technical advances of our segmentation approach. Traditional methods such as FFT-based frequency estimation or envelope peak detection rely on hand-crafted heuristics, operate on short local windows, and are easily confounded by noise or irregular respiratory [7,29]. Such methods often misidentify respiratory cycles in the presence of snoring, background noise, or variable respiratory patterns, leading to substantial errors in downstream metrics.

In contrast, the Transformer-based model utilized here, built on Whisper architecture [19], incorporates self-attention mechanisms that capture long-range temporal dependencies and complex dynamics across entire segments of sleep audio [21]. Pretrained on massive speech datasets, the model inherits robust feature representations that transfer effectively to respiratory sounds, improving resilience to environmental variability. Fine-tuning adapts these representations for precise inhale/exhale/silence classification, while majority voting across overlapping frames enforces physiologically plausible segmentation boundaries and mitigates transient noise-induced errors.

These design choices explain the superior accuracy observed in our experiments. Empirically, the proposed approach achieved substantially lowers RMSE and MAE in estimating both respiratory counts and periods compared to classical baselines, demonstrating that deep sequence models can overcome the fragility of traditional signal-processing pipelines [14,17]. Furthermore, the explicit segmentation outputs allow the derivation of clinically interpretable metrics that directly inform sleep stage estimation, bridging the gap between raw acoustic patterns and meaningful physiological insights.

Overall, the integration of Transformer-based segmentation into our framework offers a powerful, explainable, and scalable alternative to classical methods, enabling reliable capture of fine-grained respiratory patterns that are essential for sleep architecture analysis.

### 5.3. Limitations and Future Directions

This study has several limitations that should be acknowledged when interpreting the findings. First, our evaluation relied exclusively on a single public dataset (PSG-Audio). Although subject-wise cross-validation reduces optimistic bias, true generalizability across recording devices, environments, and populations remains unproven. Moreover, the cohort may not fully represent the diversity of age groups, BMI distributions, or sleep-disordered respiratory severities, raising the possibility of subgroup-specific performance differences. In addition, our analysis was based on single-night recordings, without assessing night-to-night variability or linking predictions to established clinical outcomes such as the apnea–hypopnea index, sleep efficiency, or patient-reported symptoms. Finally, real-world deployment factors—including robustness to commodity microphones, far-field bedroom acoustics, on-device latency, and privacy considerations—were not evaluated in this work. Addressing these aspects will be essential for translation into daily use.

From a technical perspective, the framework is intentionally restricted to respiratory audio. While this design emphasizes transparency and deployability, it may limit robustness in segments with weak respiratory, substantial background noise, or overlapping sounds such as snoring and speech. Incorporating complementary low-burden modalities (e.g., accelerometry, PPG, radar) could improve stability while preserving interpretability. Another limitation lies in the ground-truth labels: polysomnographic staging is known to exhibit inter-scorer variability, and our aggregation of N1 and N2 into a single Light stage may obscure physiologically meaningful differences. Finally, the pipeline is sequential, so segmentation errors can propagate to feature extraction and regression without explicit uncertainty quantification. Future work should explore uncertainty-aware prediction, label-noise sensitivity analyses, and finer-grained staging.

## 6. Conclusions

In this study, we developed and evaluated a novel, interpretable, and robust framework for estimating sleep stage proportions directly from nocturnal respiratory audio. Our modular pipeline, which incorporates precise respiratory sound segmentation, comprehensive feature extraction, and stage-specific regression models, demonstrated superior performance compared to conventional methods in respiratory analysis and yielded promising accuracy in predicting Wake, Light, Deep, and REM sleep proportions.

A key strength of our approach lies in its inherent transparency and physiological interpretability. By explicitly modeling and quantifying key respiratory dynamics, our system offers a path beyond opaque black-box predictions, providing clinically meaningful insights into how respiratory patterns may correlate with sleep architecture. This can enable a deeper understanding for clinicians and may facilitate user trust in home-based monitoring solutions. The method’s demonstrated robustness across varying respiratory patterns and its ability to generalize across subjects suggests its strong potential for practical, real-world application.

This work represents a promising step towards more accessible, contact-free, and explainable sleep health monitoring. Building upon this foundation, future research will focus on validating the system on more diverse populations, integrating multi-modal physiological signals for enhanced accuracy, and optimizing the framework for efficient, real-time deployment on consumer-grade hardware. Ultimately, this work aims to contribute to making comprehensive sleep assessment more widely accessible, supporting proactive health management and potentially improving the quality of life for many.

## Figures and Tables

**Table 1:** Hyperparameters for each regression model. Abbreviations: LR = Linear Regression; DTR = Decision Tree Regressor; RFR = Random Forest Regressor; SVR = Support Vector Regression; kNN = k-Nearest Neighbors; MLP = Multi-Layer Perceptron; TabPFN = Tabular Prior-Data Fitted Network (default pretrained weights).

Model	Hyperparameters
LR	fit_intercept=True, positive=False
DTR	criterion=’squared_error’, max_depth=None, min_samples_split=2, min_samples_leaf=1
RFR	n_estimators=100, max_depth=None, min_samples_leaf=1, bootstrap=True
SVR	C=1.0, epsilon=0.1, kernel=’rbf’, gamma=’scale’
kNN	n_neighbors=5, weights=’uniform’, metric=’minkowski’
MLP	hidden_layer_sizes=(100,), activation=’relu’, solver=’adam’, alpha=1e-4, learning_rate=’adaptive’, max_iter=1000
TabPFN	Default pretrained weights

**Table 2:** Performance of respiratory sound segmentation.

Methods	Respiratory Count		Respiratory Period	
	RMSE	MAE	RMSE	MAE
FFT	1.71	1.36	2.03	1.53
PeakFinding	4.15	3.48	2.72	2.26
**Proposed**	**1.44**	**0.96**	**1.25**	**0.81**

*Note.* Bold indicates the best performance.

**Table 3:** MAE and RMSE by fold (with averages) for different regression models across sleep stages. Abbreviations: LR = Linear Regression; DTR = Decision Tree Regressor; RFR = Random Forest Regressor; SVR = Support Vector Regression; kNN = k-Nearest Neighbors; MLP = Multi-Layer Perceptron; TabPFN = Tabular Prior-Data Fitted Network.

Model	Fold 1		Fold 2		Fold 3		Fold 4		Fold 5		Avg.	
	RMSE	MAE	RMSE	MAE	RMSE	MAE	RMSE	MAE	RMSE	MAE	RMSE	MAE
Wake												
LR	0.0698	0.0509	0.0547	0.0432	0.0503	0.0408	0.0760	0.0507	0.0844	0.0644	0.0670	0.0500
DTR	0.0981	0.0709	0.0733	0.0549	0.0893	0.0640	0.1030	0.0700	0.0937	0.0691	0.0915	0.0658
RFR	0.0618	0.0499	0.0524	0.0376	0.0502	0.0378	0.0720	0.0486	0.0671	0.0519	0.0607	0.0452
SVR	0.0792	0.0705	0.0685	0.0576	0.0681	0.0595	0.0832	0.0708	0.0815	0.0714	0.0761	0.0660
kNN	0.0650	0.0513	0.0531	0.0392	0.0541	0.0403	0.0718	0.0522	0.0659	0.0500	0.0620	0.0466
MLP	0.2525	0.1910	0.2931	0.2348	0.2761	0.2076	0.3053	0.2070	0.2966	0.2173	0.2847	0.2115
TabPFN	**0.0599**	**0.0457**	**0.0503**	**0.0370**	**0.0485**	**0.0377**	**0.0691**	**0.0452**	**0.0638**	**0.0492**	**0.0583**	**0.0430**
Light												
LR	0.1168	0.0852	0.0961	0.0769	0.0847	0.0709	0.0991	0.0801	0.1213	0.0877	0.1036	0.0802
DTR	0.1171	0.0923	0.1594	0.1226	0.1390	0.1094	0.1131	0.0859	0.1522	0.1208	0.1362	0.1062
RFR	0.0988	0.0747	0.0926	0.0727	0.0784	0.0653	0.0928	0.0739	0.1007	0.0771	0.0927	0.0727
SVR	0.1135	0.0894	0.0998	0.0837	0.0817	0.0696	**0.0908**	0.0751	**0.0949**	0.0760	0.0961	0.0788
kNN	0.1085	0.0844	0.0950	0.0742	0.0845	0.0692	0.0923	0.0722	0.1055	0.0839	0.0972	0.0768
MLP	0.2663	0.2121	0.3399	0.2839	0.3289	0.2352	0.3587	0.2261	0.3444	0.2258	0.3276	0.2366
TabPFN	**0.0981**	**0.0724**	**0.0865**	**0.0681**	**0.0720**	**0.0610**	0.0944	**0.0711**	0.0980	**0.0726**	**0.0898**	**0.0690**
Deep												
LR	0.1029	0.0631	0.0889	0.0651	0.0819	0.0578	0.1067	0.0776	0.0800	0.0561	0.0921	0.0639
DTR	0.1190	0.0610	0.1007	0.0568	0.1165	0.0715	0.1232	0.0765	0.0916	0.0625	0.1102	0.0657
RFR	**0.0859**	0.0501	0.0673	0.0454	0.0779	0.0517	0.0937	0.0667	0.0721	0.0476	0.0794	0.0523
SVR	0.1050	0.0869	0.0999	0.0908	0.0982	0.0871	0.1067	0.0982	0.0878	0.0771	0.0995	0.0880
kNN	0.0917	0.0489	0.0722	0.0467	0.0741	0.0486	**0.0884**	0.0566	0.0776	0.0509	0.0808	0.0503
MLP	0.2499	0.2024	0.2951	0.2321	0.3033	0.2314	0.3227	0.2183	0.2899	0.2162	0.2922	0.2201
TabPFN	0.0871	**0.0419**	**0.0616**	**0.0378**	**0.0671**	**0.0397**	0.0927	**0.0549**	**0.0720**	**0.0433**	**0.0761**	**0.0435**
REM												
LR	0.0732	0.0547	0.0735	0.0601	0.0608	0.0484	0.0645	0.0501	0.0769	0.0570	0.0698	0.0541
DTR	0.0703	0.0489	0.0932	0.0672	0.0700	0.0502	0.0818	0.0512	0.0886	0.0643	0.0808	0.0564
RFR	0.0618	0.0463	0.0608	0.0459	0.0443	0.0376	0.0681	0.0480	**0.0669**	0.0466	0.0604	0.0449
SVR	0.0808	0.0722	0.0790	0.0696	0.0760	0.0688	0.0724	0.0648	0.0781	0.0671	0.0773	0.0685
kNN	0.0633	0.0460	**0.0562**	0.0442	0.0502	0.0399	0.0646	0.0465	0.0715	0.0535	0.0612	0.0460
MLP	0.2227	0.1738	0.2857	0.2269	0.2638	0.2041	0.3254	0.2151	0.2932	0.2124	0.2782	0.2065
TabPFN	**0.0550**	**0.0373**	0.0578	**0.0409**	**0.0356**	**0.0288**	**0.0608**	**0.0424**	0.0710	**0.0461**	**0.0560**	**0.0391**

*Note*. Bold indicates the best performance.

**Table 4:** Accuracy within error bounds for sleep stage proportion prediction.

Error Bound	Wake	Light	Deep	REM	Average
±5%	69.37	47.18	77.46	79.93	68.49
±10%	94.01	76.06	89.79	91.55	87.85
±20%	98.59	96.13	95.42	98.59	97.18

## Data Availability

The dataset supporting the results reported in this manuscript, PSG-Audio, is a publicly available dataset. It can be accessed and downloaded from https://www.scidb.cn/en/detail?dataSetId=778740145531650048 (accessed on 7 October 2025). No additional unpublished data from this study are available for sharing, as all relevant data are either part of the publicly accessible dataset or are derived results presented within the manuscript.
